# Reply to Comments on “Shiga-Toxin Producing *Escherichia coli* in Brazil: A Systematic Review. *Microorganisms* 2019, *7*, 137”

**DOI:** 10.3390/microorganisms7100418

**Published:** 2019-10-03

**Authors:** Vinicius Silva Castro, Eduardo Eustáquio de Souza Figueiredo, Kim Stanford, Tim McAllister, Carlos Adam Conte-Junior

**Affiliations:** 1 Institute of Chemistry, Universidade Federal do Rio de Janeiro, Rio de Janeiro 21941-909, Brazil; viniciuscastro06@gmail.com (V.S.C.); carlosconte@id.uff.br (C.A.C.-J.); 2 Agronomy and Animal Science College, Universidade Federal de Mato Grosso, Cuiabá—Mato Grosso 78060-900, Brazil; 3 Nutrition College, Universidade Federal de Mato Grosso, Cuiabá—Mato Grosso 78060-900, Brazil; 4 Department of Food Technology, Faculdade de Veterinária, Universidade Federal Fluminense, Rio de Janeiro 24230-340, Brazil; 5 Alberta Agriculture and Forestry, #100-5401 1st Ave. S, Lethbridge, AB T1J 4V6, Canada; Kim.Stanford@gov.ab.ca; 6 Agriculture and Agri-Food Canada, Lethbridge Research and Development Centre, 5403 1st Avenue South, Lethbridge, AB T1J 4B1, Canada; Tim.McAllister@agr.gc.ca; 7 National Institute of Health Quality Control, Fundação Oswaldo Cruz, Rio de Janeiro 21040-900, Brazil

Recently a comment regarding our article entitled “Shiga-Toxin Producing *Escherichia coli* in Brazil: A Systematic Review” was made by Dr. Guth. We would like to reaffirm our hypotheses and conclusions, which were obtained by evaluating articles published in the literature between 2000 and 2018. The relevant literature is highlighted in Figure 3 of the article, and show that no data has been collected in 44% of Brazilian states. Proposing epidemiological data for the whole of Brazil, we ignored the presence of several variables, such as the principles of statistical sampling, borders with 10 other countries, difference between the types of animals raised, vegetation type, different temperature ranges, and cultural differences.

Firstly, we were happy to note a concern about this emerging issue that has been thoroughly investigated worldwide. For this reason, the authors were motivated to perform the first systematic review on this topic in the country [1]. To do this, we compiled data from articles published only in scientific journals, with a focus on the entire territory of Brazil. The focus of the review was to describe the serotypes reported in three different matrices (animal, food and human infection) and to investigate the distribution of reported cases among the states of the entire country.

Recently, the author Guth wrote about some concerns about our article. The first point addressed was in relation to our statement: “*Although no human disease outbreaks in Brazil related to STEC* [Shiga-toxin producing *Escherichia coli*] *has been reported ...*”. In fact, in the study mentioned and conducted by Vaz et al. [2], it was identified that “*indistinguishable PFGE* [pulsed-field gel electrophoresis] *pattern for two O157:H7 STEC strains were isolated in the same geographic area at an interval of approximately 15 days* …”. However, if we follow the definition of the Ministry of Health, which is the official organ of the country, foodborne outbreaks are defined as an: “*Episode in which two or more people show the same symptoms after ingesting food and/or water from the same source*” (Translated) [3]. Considering this concept, we observed that Vaz et al. [2] did not record information about possible sources of food or water that could be associated with or could have caused a possible O157 outbreak in Brazil. In addition, although the article suggests that an outbreak may have occurred, this suggestion does not correspond to a confirmation. This fact is supported by the following sentences in the discussion of the paper: “*first occurrence of an O157:H7 outbreak in Brazil during that period **can be** suggested* [2]” and “*These results **may be** interpreted as an **indication** of the first **possible** O157:H7 outbreak in Brazil*” [2]. Therefore, due to the impact of a food-related outbreak, being liable for legal damages totaling $50 million in a single outbreak case [4] and not finding an official confirmation, we understand that it is correct to say that there is still not a confirmed foodborne outbreak of STEC in Brazil.

Regarding Guth’s contention that the following hypotheses in our manuscript are incorrect: “(i) disease outbreaks are not recorded due to a lack of a centralized reporting system or (ii) disease outbreaks are not being recognized as there is no surveillance system for STEC” [1], in line with the whole article, these hypotheses refer to the entire country of Brazil. We are aware that the state of São Paulo launched the Monitoring of Acute Diarrheal Diseases (MDDA) [5] system and surveillance of outbreaks of DTA, which we hope will branch out to other states. In time, we hope that there will be a centralized reporting system specifically for STEC in Brazil, since the Ministry of Health’s Portaria No. 1984 refers to compulsory reporting of hemolytic uremic syndrome (HUS) [6]. However, according to the World Health Organization, only 10% of cases of STEC infection develop HUS [7]. In addition, HUS is not exclusive to STEC, as *Shigella* spp. also have the ability to cause HUS [8,9]. Therefore, we reaffirm our assumptions (i) **or** (ii), with particular reference to the fact that 10 states plus the federal district (44% of the entire country) have not yet published data on STEC (Figure 3) [1]. 

With relation to the sentence: “*The authors concluded that “O157:H7 serotype had the highest occurrence rates in animal, food and humans in Brazil, and that this higher prevalence might be related to its ease detection. Unfortunately, these conclusions cannot be confirmed by the data*”. This statement needs to be associated and contextualized with the review’s phrase: “*It can be readily isolated as non-sorbitol fermenting colonies (the main O157 characteristic)* [1]”. Moreover, our statement is supported by the work of Verhaegen et al. [10], which also indicated that O157:H7 was the most common serotype within the STEC group initially, and that the development of isolation media has been targeted towards this serotype. Another point is that, even today, articles are published proposing an investigation into the cultivability of non-O157 [10,11,12]. Furthermore, in the review performed by Bettelheim in 2007 [13], an important finding was presented: “Unfortunately, at present, there are no specific media for non-O157 STEC”. Since 2007, this problem has still not been solved. These articles provide evidence for why serotype O157:H7 has been verified in all matrices. However, we did not conclude that better media for O157:H7 was the cause. Instead, we presented a hypothesis when discussing the results, as seen in the sentence: “*Consequently, a higher prevalence of O157:H7 in Brazil **might also be related** to its ease of detection* [1]”.

In relation to the study performed by Paula and Marin [14], serotype O157:H7 was not detected. Thanks for this clarification. We apologize and will request an erratum to the editor to correct this point. For the specific case of cheese samples in the work carried out by Carvalho et al. [15], wherein *stx* genes were not amplified, strains that were considered positive following detection by the VIDAS^®^ ECO O157 kit—which involved a pre-enrichment step of MacConkey broth with cefixime and potassium tellurite—were later plated in Chromo Agar^®^ for confirmation via a serum agglutination test for O157:H7. We believe that, in spite of the fact amplification of *stx* genes was not performed, the work followed a scientific methodology that supported the isolation of this strain. In addition, the study represented the only food research in the Goiás state. For these reasons, it was included in our review. The same understanding was attributed to the Panetto article [16], which followed the STEC serotyping protocol despite not amplifying *stx* genes.

Regarding the following points cited by Guth: “*another relevant correction to be made is that O157:H7 serotype is the most frequent serotype associated with more severe infections such as HUS and hemorrhagic colitis, but if we consider human infections as a whole non-O157 serotypes such as O111:H8, O103:H2, O118:H16 and several others are those most frequently identified*…”. We thank the author for this observation, as well as the references cited. However, in the review work, our focus was to exclusively analyze STEC epidemiological reports from the last 18 years with a basis only in papers published in scientific periodical journals—eBooks were not considered [17]. In addition, our affirmation that the “*O157:H7 serotype is the most frequent serotype associated with more severe infections such as HUS and hemorrhagic colitis*”, can be also supported. Ori et al. [18] found “*HUS cases were only associated with STEC serotype O157:H7*”. Regarding the article by Leomil et al. [19], the serotype O118:H16 was isolated from fecal samples of cattle and is listed in Table 1, but in the text it is only cited in human. Thanks for the indication, we apologize and will request an erratum to the editor to correct this point.

We would also like to discuss this sentence by Guth about the conclusion of our paper: “*Castro et al. [1] claimed that the prevalence and distribution of STEC serogroups in Brazil remains unclear. Certainly, this statement cannot be considered as true, otherwise all the survey conducted by the authors and shown in Tables 1–3 would not have been possible*”. In the conclusion of our paper [1], we emphasized the need for improved epidemiological monitoring because 10 states and the federal district (44% of the entire country) have not published scientific articles on STEC epidemiology. Moreover, if we had considered the states that had not published work in one of the matrices analyzed (animal, food or human), this number would have increased to 88%; i.e., 22 out of 25 states in Brazil lack information about STEC. If we made an epidemiological description of the country, ignoring this point, we would be taking the results from the southeast and south of the country and extrapolating them to the entire national territory, even though the purpose of the review was to consider STEC epidemiology in all of Brazil. In addition, we would be neglecting several variables, such as statistical sampling principles, the 10-country boundary, and the difference between the types of animals raised, vegetation type, different temperature ranges, and cultural and regional differences.

In conclusion, we reinforce the hypotheses suggested in the text and the need for further research on STEC contamination in Brazil, especially in the north, northeast and midwest of the country. 
